# Mutation Size Optimizes Speciation in an Evolutionary Model

**DOI:** 10.1371/journal.pone.0011952

**Published:** 2010-08-03

**Authors:** Nathan D. Dees, Sonya Bahar

**Affiliations:** Department of Physics and Astronomy and Center for Neurodynamics, University of Missouri at St. Louis, St. Louis, Missouri, United States of America; University of Zurich, Switzerland

## Abstract

The role of mutation rate in optimizing key features of evolutionary dynamics has recently been investigated in various computational models. Here, we address the related question of how maximum mutation size affects the formation of species in a simple computational evolutionary model. We find that the number of species is maximized for intermediate values of a mutation size parameter μ; the result is observed for evolving organisms on a randomly changing landscape as well as in a version of the model where negative feedback exists between the local population size and the fitness provided by the landscape. The same result is observed for various distributions of mutation values within the limits set by μ. When organisms with various values of μ compete against each other, those with intermediate μ values are found to survive. The surviving values of μ from these competition simulations, however, do not necessarily coincide with the values that maximize the number of species. These results suggest that various complex factors are involved in determining optimal mutation parameters for any population, and may also suggest approaches for building a computational bridge between the (micro) dynamics of mutations at the level of individual organisms and (macro) evolutionary dynamics at the species level.

## Introduction

Why do mutation rates not evolve to zero? It is now more than seventy years since A. H. Sturtevant posed this question [1], raising a problem in evolutionary biology which remains unresolved. Some pieces of the puzzle are emerging, however, from experimental and, increasingly, from computational studies. Arguments from biochemistry and bioenergetics suggest the existence of a physicochemical *lower* bound on how far mutation rates can be decreased [2]. A related, though not identical, lower limit is likely imposed by the “cost of fidelity”, the combined metabolic and temporal cost of reaching for perfection in replication and transcription fidelity [2], [3]. Beyond a certain limit, an organism would expend an amount of energy on proofreading that would not be worth the minimal gain in fidelity, a limit which might be most aptly described by the old adage “the best is the enemy of the good”.

The origins of limits on mutation rates *from above* are perhaps harder to untangle. A source of variation is obviously necessary for the process of natural selection. On the other hand, too high a mutation rate has obvious negative consequences [4]–[6]. Is there, then, an *optimum* amount of variability?

Drake's studies of microbial genetics showed similar mutation rates across a wide range of genome sizes [7], [8]. However, as with any biological problem, exceptions to the rule were quick to follow, and evidence for a universal mutation rate in eukaryotes has not been forthcoming [2]. Other studies suggest that observed mutation rates, whether optimal or not, are certainly not minimal. Studies in bacteriophage T4 [9], [10], E. coli [10] and Drosophila [11] have demonstrated that mutation rates can be driven lower than (or can increase above [12]) wild type values under various external pressures, and can be restored to wild type when control conditions are reestablished [12]. Evolutionary stress can drive selection for increased mutation rates: mutator bacterial strains are more antibiotic resistant than non-mutator strains, and thus have a clear selective advantage, potentially leading to an increase in the overall mutation rate within a population [13]. Mutation rate variability is a key theme in current discussions of the need for an extended evolutionary synthesis [14], [15] under the name of “the evolution of evolvability”, the tantalizingly recursive possibility that the ability of organisms to evolve is itself a trait, or spectrum of traits, under evolutionary control [16]–[19].

The question of mutation rate optimization is compounded by the problem of causality, to the extent that an increased mutation rate cannot be selected for in a *current* organism on the basis of that organism's *descendants'* increased ability to radiate into new ecological niches. In other words, the fact that a higher mutation rate will help later generations does not explain how it can be selected for in the current generation, for which it does not have a clear advantage. As a result, mutations in genes that control mutation rate may often hitchhike along with other mutations that confer immediate selective advantage [2], [16]. This sort of hitchhiking is obviously less prevalent in organisms with more genetic recombination [2].

Several recent computational studies have addressed the problem of optimum mutation rate. Three of these studies, Bedau and Packard [19], Earl and Deem [16] and Clune et al. [20] specifically address the problem of *whether an intermediate mutation rate can optimize fitness*. In the present paper, we investigate a closely related problem, the *optimization of the number of species as a function of maximum mutation size*. Since we pose similar questions and take a similar approach to those of the three papers just cited – though in the context of speciation rather than of individual fitness – we briefly review each of those works.

Bedau and Packard [19] investigated optimal mutation rate in the context of a “balance [between the] competing demands” of evolutionary *novelty* (adaptability) and evolutionary *memory* (adaptedness). They explored the optimization of fitness as a function of mutation rate in a model of reproducing agents on a toroidal lattice; the agents consumed energy from a “continually augmented external source”, and reproduced when they had accumulated sufficient resources to split into two organisms. The organisms were characterized by their strategies of interacting with the environment and gathering resources; their fitness, defined as the amount of food gathered, was found to be optimized for an intermediate mutation rate, where the mutation rate represented the rate at which strategy elements were selected from a pool of possible behaviors. Bedau and Packard also performed simulations *in which agents with different mutation rates competed against each other*; they found that those with a specific intermediate mutation rate were the most successful. Using measures of diversity, they also demonstrated that “the mutation rate governs a transition between two qualitatively different phases of evolutionary dynamics”, namely a more ordered state characterized by long periods of stasis for low mutation rates, and highly disordered dynamics where “the gene pool tends to be a continually changing plethora” of strategies at high mutation rates [19]. The optimal mutation rate marked the boundary between these two phases, and was suggested by the authors to indicate a possible adaptation to “the edge of genetic disorder”, implying a close relation between this result and other studies of phase transition-like behavior in complex systems, where complex behavior is found to exist at the boundary between regimes [21], [22].

Earl and Deem [16] took a different approach entirely, investigating the minimization of an energy function in a model of protein evolution. Their simulated proteins experienced point mutations as well as larger recombination/swapping events. The rates of both types of mutations could be selected for. Fitness was determined by the minimization of an energy function involving subdomain interaction energies and binding energies. Protein evolution took place on a fitness landscape determined by various properties of the environment; the landscape was shifted periodically. Earl and Deem found that larger mutational shifts (recombination/swapping rather than point mutations) were selected for, and became dominant, when landscape shifts occurred faster and/or were larger. This result demonstrates first that, in this model, as in that of Bedau and Packard, evolvability can be selected for. Equally important, Earl and Deem's study showed that different mutation rates can be selected for in different environments; this implies that there is not necessarily an optimum mutation rate, but rather that *different evolvability characteristics can be optimal under different circumstances*. This result was already suggested by the bacteriophage and Drosophila studies cited above; it appears again in another computational evolutionary model, that of Clune et al. [20].

Clune et al. [20] explored the optimization of mutation rate using the Avida model, in which computer programs compete as digital organisms, with their success at performing certain computational operations serving as a measure of fitness. Fitness was measured over a range of mutation rates, and was found to be maximized at an intermediate value. Next, Clune et al. allowed various digital organisms, with various values of the mutation rate, to compete against each other. When the organisms competed on a smooth landscape, the surviving organisms exhibited a mutation rate close to the optimal intermediate value. However, when the organisms competed on an irregular, rugged landscape, the surviving mutation rates were significantly lower than the optimal value, in contrast to the results of Bedau and Packard. This work again suggests that different mutation rates may be optimal under different circumstances; moreover, it suggests that even if a value *is* optimal, it may not be *reachable* via a natural selection algorithm operating on a highly rugged landscape. The authors interpret this in terms of the short term vs. long term cost of mutation rates: a high mutation rate would provide the benefit of rare large mutations that could “carry the organism over a valley to the next fitness peak”, but would also exact a high energetic cost due to the occurrence of mutations that are “not quite large enough”, costing the organism dearly, but leaving it stranded in the valley it was trying to escape. Clune et al. contrast their results with studies suggesting that high mutation rates can be optimal in all circumstances, commenting that “it seems unlikely that stably high mutation rates, such as those for RNA viruses, are maintained primarily because of the rapid adaptive capacity they bestow, as has sometimes been argued” [20].

The three studies summarized above address the optimization of a measure of individual organismal fitness. However, individual fitness is not the only quantity that can be optimized by natural selection. Darwin himself explored the idea that diversity itself may be selected for, and that phyla that are better at radiating may also be better at flourishing. This can be envisioned as an optimal filling of morphospace or, in more nineteenth-century terms, as a “Benthamite optimization calculus” [23]. These questions are deeply complicated by – and may also be critical to – the ongoing discussion of the various levels at which natural selection operates, and the interplay between these levels. Here, we investigate the optimizing role of a mutation parameter in a spirit similar to the three studies described above; however, instead of focusing on individual fitness, we address the optimization of the number of species, represented in our model as clusters of organisms in a simulated morphospace.

## Methods

The motivation for the design of the present model, implemented in MATLAB, was to incorporate the three fundamental aspects of Darwinian evolution, *variability, heritability and overpopulation*, in the simplest possible manner. Organisms exist in a two-dimensional morphospace, where each axis represents a hypothetical phenotype. At each time step, a new generation of organisms is produced via an assortative mating algorithm. The number of new organisms depends on an underlying fitness landscape; the locations of new organisms in the morphospace are determined by the locations of their parent organisms, as well as by the mutation size. We investigate the clustering of organisms, where clusters are defined as reproductively isolated groups and serve as an analogue of species, as a function of maximum mutation size.

### Organisms within a morphospace

**Figure 1:**
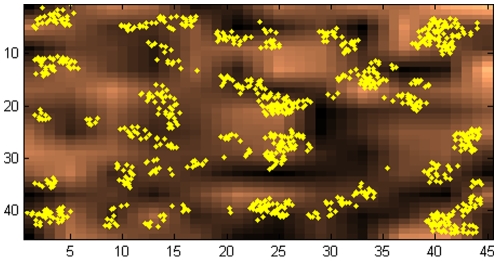
Results of a simulation after 1000 generations. Diamonds show the location of individuals in the morphospace; the color scale indicates the fitness levels corresponding to each location in the morphospace. In this realization of the model, μ = 0.88, the landscape is influenced by feedback from the population density, and the mutation sizes are normally distributed.

Simulated organisms exist on a landscape in a two-dimensional morphospace, with the x- and y-coordinates corresponding, respectively, to a given organism's two traits. This is illustrated in Figure 1; diamonds show the locations of organisms within the space. In this implementation of the model, the landscape axes range from 0 to 45; organisms cannot exist beyond the boundaries, i.e., the morphospace is finite. Note that the simulation could be performed with a morphospace of variable size and with different boundary conditions; note also that the landscape axis units are arbitrary.

### Assortative mating

The model uses assortative mating, whereby, in each generation, every organism picks the nearest other organism in the landscape and mates with it to produce new organisms for the next generation. The choice of an assortative mating scheme is motivated at once by its simplicity and its realism. Recalling that the organisms exist in a morphospace rather than a physical space, it should be immediately apparent that the simplest realistic mating scheme is one in which phenotypically similar organisms mate with each other rather than with more phenotypically distant organisms.

Assortative mating schemes have been extensively used in various studies, such as investigations of the mechanisms of sympatric [24] and competitive [25], [26] speciation. Given what de Cara et al. [27] describe as “the ubiquity of assortative mating in nature”, other recent studies have focused on the evolution of assortative mating itself [27]–[29]. It should be emphasized that clustering of organisms is by no means a given outcome of assortative mating. As recently as 1995, Maynard Smith and Szathmáry commented that “it is plausible that a ‘sexual continuum’, in which there are no discrete species and individuals can mate with others not too distant from themselves, would break up into species… However, we are not aware of any explicit model demonstrating the instability of a sexual continuum” [30]. The conditions under which such clustering occurs are a central focus of the present investigation.

### Generation of new organisms

If (*c*_1*x*_,*c*_1*y*_) and (*c*_2*x*_,*c*_2*y*_) are the morphospace coordinates of the two parent organisms, the coordinates (*c_bx_*,*c_by_*) of a next-generation organism are given by
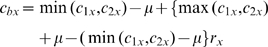
(1a)

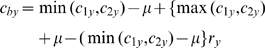
(1b)where 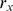 and 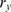 are random numbers selected from a *uniform distribution* between 0 and 1, and where μ represents the maximum possible mutation size.

In another version of the experiment, the coordinates (*c_bx_*,*c_by_*) of a new organism are calculated as follows:
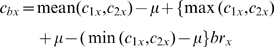
(2a)

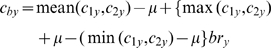
(2b)with 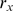 and 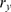 selected from a *normal distribution* with zero mean, and *b* = 0.1581. Again, μ represents the maximum mutation size. Thus, for both versions of the model, the coordinates of each new organism are randomly chosen to lie within a range defined by the coordinates of the parents, but with the boundaries of the range extended by the parameter μ. Note that μ can be easily related to a mutation rate *M* by integrating the rate over (generation) time. Thus,
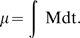
If *M* is constant, then the maximum mutation size μ will be directly proportional to the mutation rate *M*.

### Underlying fitness landscape

The landscape, in addition to having two dimensions indicative of trait values, also has a third dimension, which, when visualized, resembles the elevation of the space (see the morphospace color scale in Figure 1). The elevation at any location on the landscape represents the fitness level available to organisms residing at that location. These fitness levels, ranging from 1 to 4, are realized in the model as the number of offspring an organism will produce.

The fitness landscape originates from a randomly-generated 12×12 matrix of fitness levels. Linear interpolation is used to expand the matrix to dimensions 45×45. After the initial distribution of fitness levels is generated, the fitness landscape changes during the simulations in one of two different ways, either (1) shifting gradually or (2) being altered by feedback from the local density of organisms.

For the randomly shifting landscape, every λ generations, the last column is deleted from the 12×12 random matrix underlying the fitness landscape. The other n columns are shifted to the *n+1* position, leaving a “hole” at the first column of the matrix, which is replaced by 12 new, randomly-generated values, after which interpolation is performed again to generate a 45×45 landscape. These operations have the effect of shifting the landscape gradually to the right. The parameter λ was set at 2 throughout the simulations shown here.

For landscapes modulated by feedback, in every generation, the fitness value at each location in the landscape grid was decreased by an amount proportional to the number of organisms living in the region. For the models implemented here, the proportionality was set at 0.0071. These reactive changes in the landscape symbolize the depletion by over-use of the available resources in a given ecological niche. The total summation of fitness values available across the entire landscape was conserved in each generation; this was done by adding back the entire subtracted quantity after dividing it equally amongst each of the 144 elements of the fitness landscape matrix (before interpolation). Through this method, areas in the morphospace which were unaffected by the subtraction (areas whose resources were not depleted) become increasingly advantageous for reproduction.

### Random death and overpopulation

In order to introduce further randomness into the model, a fraction ρ of the new organisms are randomly eliminated before the start of the new generation, where, for each generation, ρ is a random number selected from a uniform distribution between 0 and 0.70. The effects of overpopulation are implemented by setting a distance limit within which only one organism can exist. In all the implementations of the model shown here, the overpopulation limit is set at 0.25.

### Competition among organisms with different values of μ

In order to investigate the interactions between organisms with different mutation parameters, the model was modified so that each organism was assigned not only coordinates in the morphospace, but also a distinct value of μ (which was held constant over the entire population in all other simulations). The mutation parameter μ for each individual in the initial population was selected randomly from a uniform distribution between 0 and 1. Note that the choice of a maximum value of μ = 1 is motivated solely by the range of μ values used in other simulations shown here; there is no a priori limit for the value of μ.

The simulation was performed identically to the other simulations (using the model with a shifting, rather than a feedback-modulated, fitness landscape, and with uniformly distributed mutation values within the limits set by μ), except that each new organism takes the μ value of one of its parents.

### Clustering algorithm

Clusters, the analog of species in our model, are determined by who mates with whom. The development of this algorithm was motivated by the concept of *biological species*, in which species are defined as reproductively isolated groups, i.e., groups with the *ability* to interbreed, developed by Dobzhansky, Mayr and others in the early days of the modern synthesis [31]. A similar species definition was also used in another recent computational study, that of de Aguiar et al. [32].

As implemented here, the clustering algorithm is carried out as follows. For a given organism in a given generation, a search is performed to find all the organisms that it, as well as its nearest neighbor (its mate) and its second-nearest neighbor, have mated with. Then a similar search is performed for each of the organisms found during this first search. This iterative search continues until a closed set – a cluster – is obtained, where all organisms within the set have mated, in that generation, only within the set. This algorithm assigns each organism to one, and only one, cluster, and arrives at a unique solution for each generation.

It should be clarified that the definition of clusters implemented here is based on “who does mate with whom” rather than “who can mate with whom”, and thus we have described it as being *inspired* by, rather than being an explicit implementation of, the biological species concept. Several points need to be mentioned in this regard.

First, let us consider the extent to which our cluster definition *can* be interpreted as defining “who can mate with whom”. Consider one individual in the cluster, and its mate. A third organism which mates with the mate is also included in the cluster, and therefore the first individual chosen to seed the cluster *could* presumably mate with this third individual, under an expanded version of our assortative mating criterion. To this extent, we do indeed implement a criterion of “who could mate with whom”.

A second point to be emphasized is that a more explicit implementation of the rigorous definition of biological species would necessitate a *top-down* definition of clusters (for example, specifying that individuals could mate with organisms within a given radius). Such a top-down definition would undermine the crux of the approach taken here, which is to *capture fundamental dynamical features which emerge naturally from a model satisfying certain basic criteria of evolving systems*.

### Implementation of the model

After all the parent organisms in a given generation have produced a new generation of organisms and some of the new organisms have been culled, the parent organisms vanish and a new generation begins, with the previous offspring now playing the role of parents. In the implementation of the model used here, the initial generation consisted of 300 individuals randomly placed within the landscape; during subsequent generations the population fluctuated between several hundred and nearly ten thousand organisms.

Five experiments were performed: (1) uniformly distributed mutations with shifting fitness landscape, (2) uniformly distributed mutations with feedback-modulated landscape, (3) normally distributed mutations with shifting fitness landscape, (4) normally distributed mutations with feedback-modulated landscape, and (5) competition between organisms with different maximum mutation parameters (μ) on a shifting fitness landscape with uniformly distributed mutation values within the limits set by μ.

Experiments (1)–(4) were run for a range of values of μ, with all other parameters held constant. For each experiment and at each value of μ, the simulation was allowed to run for 1000 generations; in some cases, the population became extinct before 1000 generations were reached. Over the course of each simulation, various parameters were recorded at each generation, such as the total population size, the number of clusters, the mean distance between individuals in a cluster, etc. For each experiment, five runs were performed at each value of μ.

## Results

The results of a typical simulation after 1000 generations are shown in Figure 1. As described above, the shaded background of the landscape corresponds to the fitness level of individuals at that location, with individuals in the darkest regions being the least fit (one offspring each), and those in the lightest regions being the most fit (four offspring each). In the first generation, 300 organisms were randomly seeded throughout the landscape with a uniform distribution. By the end of 1000 generations, as shown here, organisms occur in clusters throughout the landscape. In this realization of the model (discussed in more detail below), there is negative feedback between the population and the fitness levels available on the landscape, so that when many organisms grow in the most advantageous regions, the regions' underlying fitness levels decrease. This leads to clustering along the boundaries between the regions offering the highest and lowest fitness.

**Figure 2:**
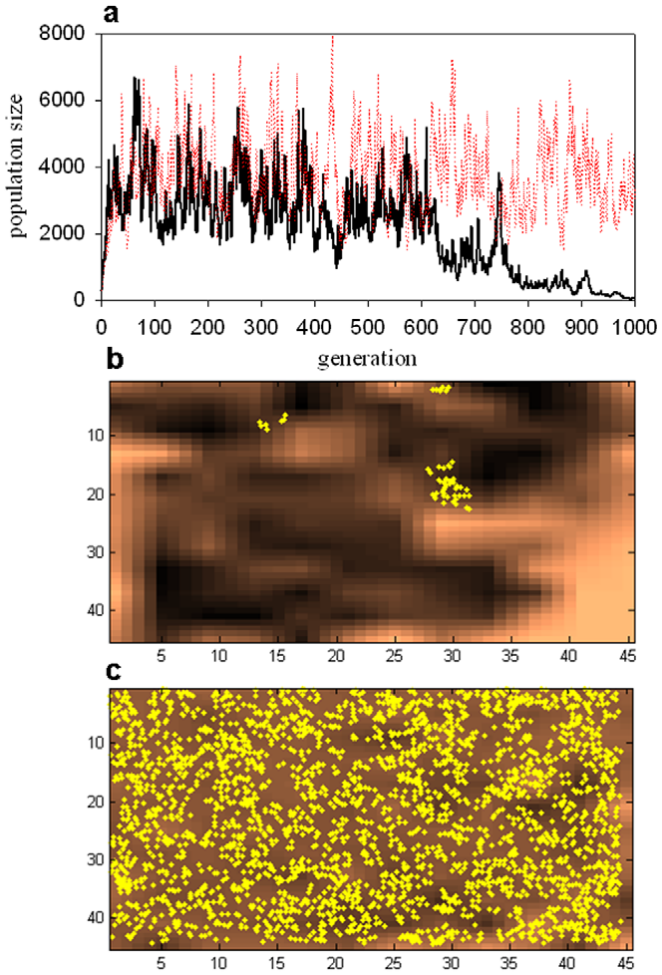
Historical contingency. (a) Population size vs. number of generations for two runs of the simulation under identical conditions (μ = 0.9, mutations normally distributed, landscape modulated by feedback). (b) Simulation at generation 1000 for run 1 (solid line in 2a), showing near-extinction. (c) Simulation at generation 1000 for run 2 (dotted line in 2b), showing a large population spread throughout the landscape.

In some cases, simulations with identical parameters exhibited a high degree of historical contingency, as illustrated in Figure 2. Here, two simulations, with μ = 0.9, mutation values selected from a normal distribution, and a feedback-modulated landscape, show dramatically different outcomes. Figure 2a shows the population for each of the two runs as a function of generation. For one simulation, the population fluctuates and then suddenly plummets nearly to extinction, while the population in the other simulation continues to fluctuate without crashing. Snapshots of the two simulations at generation 1000 are shown in Figures 2b and 2c.

**Figure 3:**
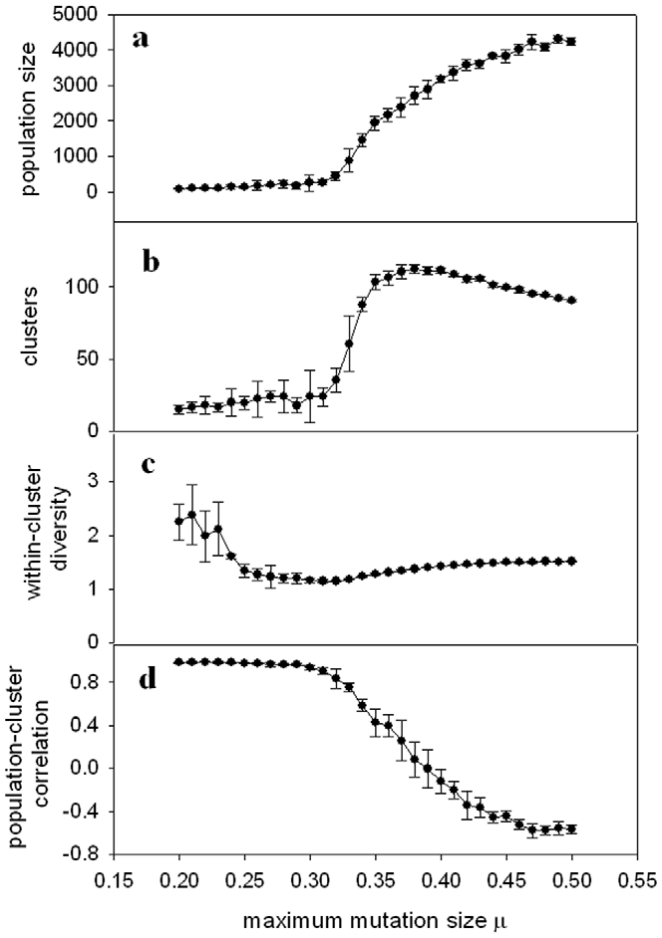
Shifting landscape model with uniformly distributed mutations. (a) Population size, (b) Number of clusters (species), (c) Mean distance between individuals in a cluster (diversity), and (d) Correlation between number of clusters and population size, all shown as a function of μ. Symbols show mean values over five realizations of the simulation at each value of μ; error bars show standard deviation among five realizations. Other parameters are given in the text.

Figure 3 shows results for the shifting landscape model, with mutation values distributed uniformly within the limits set by μ. Figure 3a shows the mean population size as a function of μ, averaged over all generations where the population size exceeded zero. At each value of μ, the mean population size is averaged over five realizations of the simulation. The population size remains small for low values of μ, and then begins to rise sharply for intermediate values, before reaching a plateau. For low μ, the populations typically tend toward extinction, and survive for more generations as μ increases.

In Figure 3b, we show the number of clusters as a function of μ. The clusters behave similarly to the population size, with one crucial exception: instead of reaching a plateau, they reach a maximum and then begin to decrease for the largest values of μ. Thus, a maximal number of clusters (species) is achieved for an intermediate value of μ.

In order to obtain a measure of the diversity within species/clusters, we calculated the mean Euclidean distance in the morphospace between individuals in a cluster. This value was averaged over all clusters and all generations to give the within-cluster diversity at a given μ. This diversity measure is shown, averaged over five realizations of the model at each value of μ, in Figure 3c. Unlike the population size and the number of clusters, the within-cluster diversity shows a gradual decline, reaching a minimum for values of μ just preceding the sharp increase in the mean population size and the number of clusters, and then gradually rising.

Figures 3a and 3b suggest that, for low values of μ, the mean population size correlates with the mean number of clusters. Correlation between these two quantities is also observed within each run of the individual simulations as well, as shown in Figure 3d by the correlation coefficient between *the time series of the population size* and the *time series of the number of clusters*. At values of μ for which the population size and number of clusters sharply increase, however, the correlation coefficient drops, and also shows an increase in variability from one simulation run to another, indicated by the increased standard deviation. For some runs, there is a positive correlation between population and clusters; for others, a negative correlation. As μ reaches values corresponding to the population plateau, a consistent anticorrelation is observed between the two quantities.

**Figure 4:**
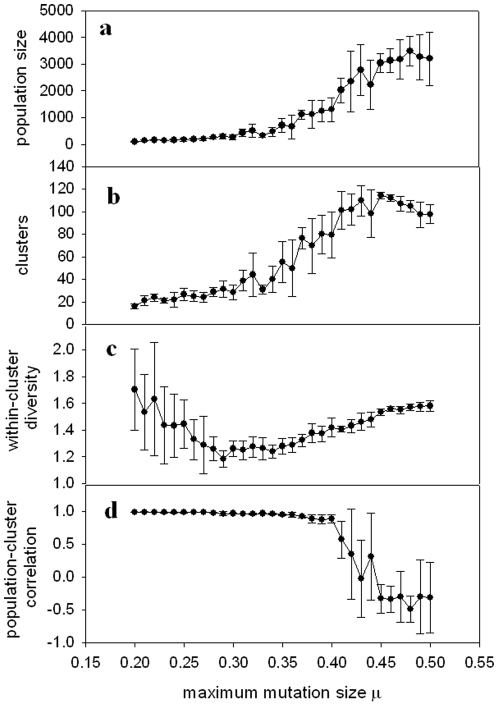
Feedback-modulated landscape model with uniformly distributed mutations. (a) Population size, (b) Number of clusters (species), (c) Mean distance between individuals in a cluster (diversity), and (d) Correlation between number of clusters and population size, all shown as a function of μ. Symbols show mean values over five realizations of the simulation at each value of μ; error bars show standard deviation among five realizations. Other parameters are given in the text.

**Figure 5:**
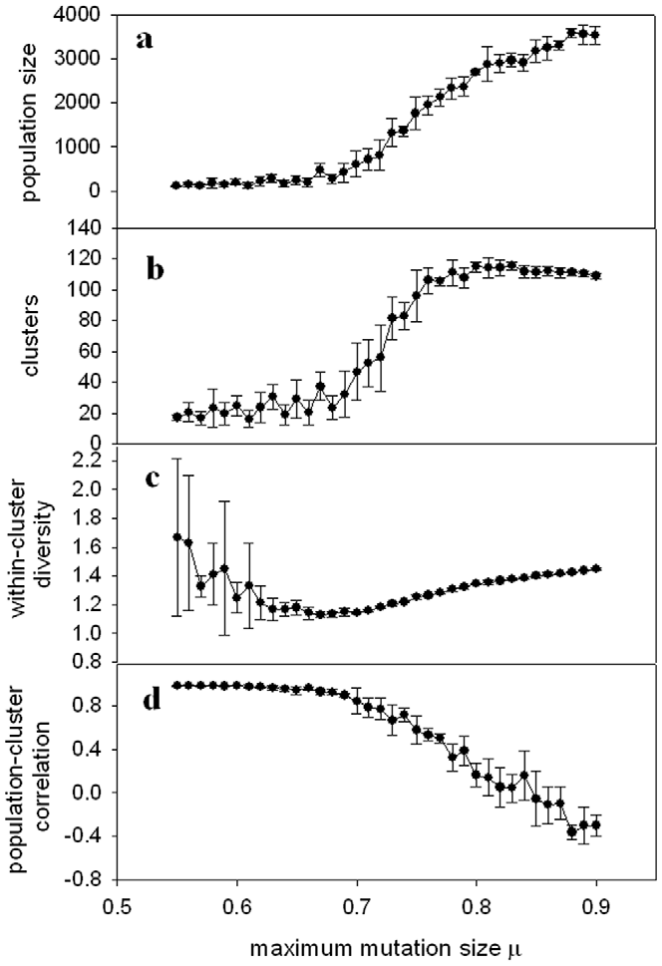
Shifting landscape model with normally distributed mutations. (a) Population size, (b) Number of clusters (species), (c) Mean distance between individuals in a cluster (diversity), and (d) Correlation between number of clusters and population size, all shown as a function of μ. Symbols show mean values over five realizations of the simulation at each value of μ; error bars show standard deviation among five realizations. Other parameters are given in the text.

**Figure 6:**
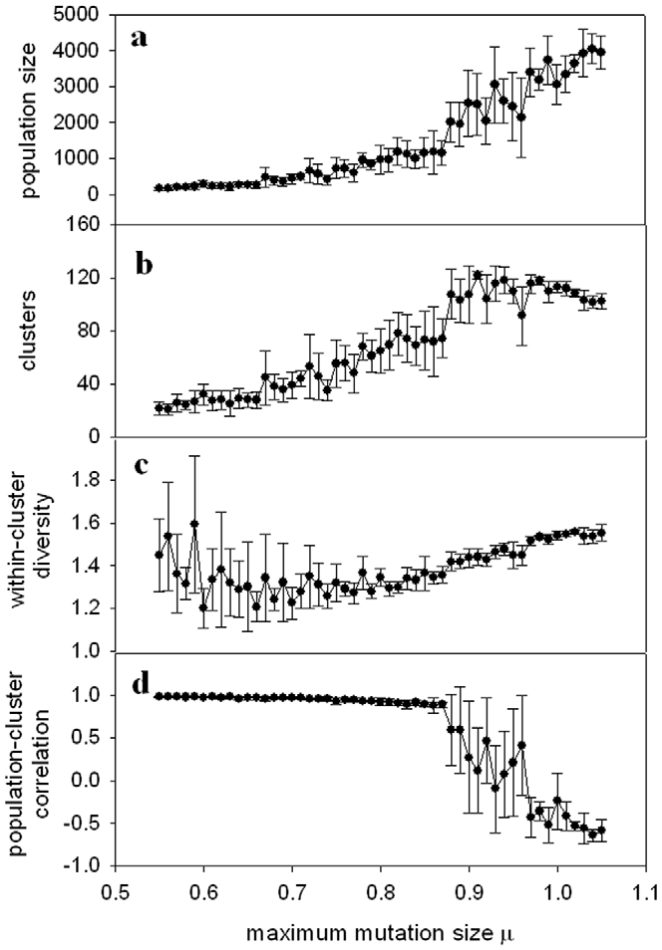
Feedback-modulated landscape model with normally distributed mutations. (a) Population size, (b) Number of clusters (species), (c) Mean distance between individuals in a cluster (diversity), and (d) Correlation between number of clusters and population size, all shown as a function of μ. Symbols show mean values over five realizations of the simulation at each value of μ; error bars show standard deviation among five realizations. Other parameters are given in the text.

Figures 4, 5 and 6 show (a) population size, (b) number of clusters, (c) within-cluster diversity and (d) correlation between population size and number of clusters, for three other implementations of the model (experiments 2–4 as defined above). The results from these various modified versions of the model are consistent with the results shown in Figure 3. The population size tends toward a plateau, while the number of clusters reaches a peak and then falls off. Within-cluster diversity reaches a minimum for values of μ immediately preceding the sharp rise in population size and number of clusters. Population size correlates positively with number of clusters for μ values below that for which the sharp population increase occurs, followed by a precipitous drop in the correlation coefficient around this “critical” range of μ.

**Figure 7:**
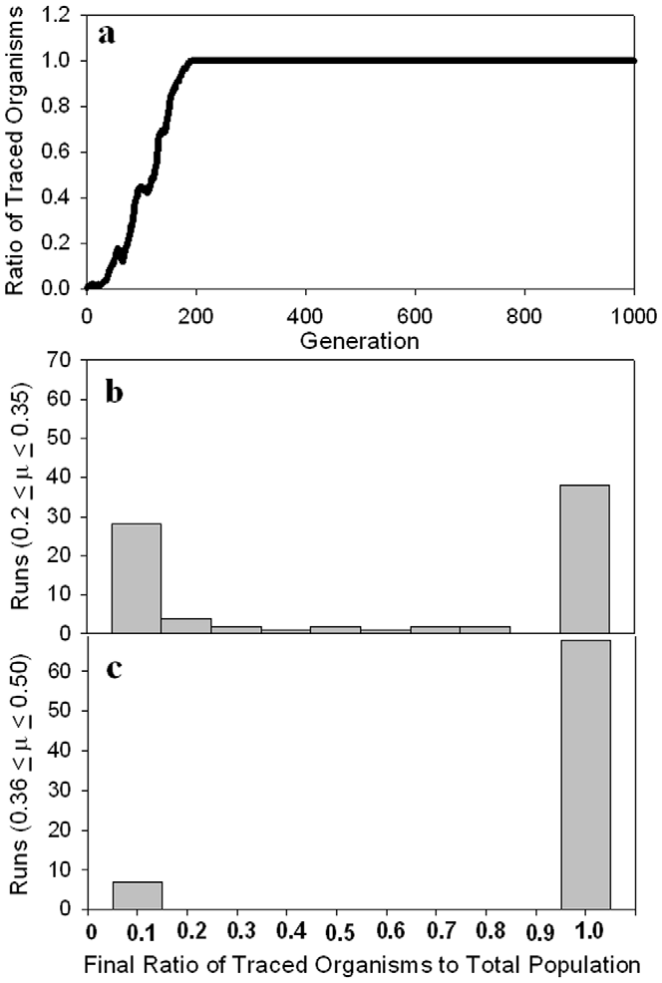
Spread of a genealogy through the population. Results are shown in the case of a shifting landscape with uniformly distributed mutation values (same conditions shown in Figure 3). (a) Ratio of descendants of the initial labeled organism to the total population, as a function of time (in units of generations), for one trial with μ = 0.5 (b and c). Histograms bin trials according to the maximum ratio of organisms descended from a single, random individual, for (a) 0.2≤μ≤0.35 and (b) 0.36≤μ≤0.5.

In order to investigate the spread of an organism's descendents through the population, a random organism of the initial generation was labeled, and its descendents traced through subsequent generations. The maximum ratio of labeled organisms to total organisms is illustrated in Figure 7a. In many cases, like the one shown in this example, the traced organisms ultimately constituted 100% of the population, indicating that all the organisms can count the original labeled organism as an ancestor. In many other cases, however, the descendants of the labeled organism died out quickly, never constituting more than a tiny fraction of the population. Figure 7b illustrates a histogram of the ratio of traced organisms to total population, compiled over all simulations for μ≤0.35. Figure 7c shows a histogram for μ≥0.36. Note that for larger values of μ (Fig. 7b), the labeled organism's descendents are more likely to spread through the entire population. To correctly interpret this result, it is important to emphasize that the survival of one organism as an ancestor *does not preclude* others from doing the same. A traced organism has the original labeled organism as *one* of its ancestors, but this does not imply that this was its *only* ancestor. The fact that the final fraction of traced organisms increases with μ is likely a result of the increased mixing of the population as μ increases.

**Figure 8:**
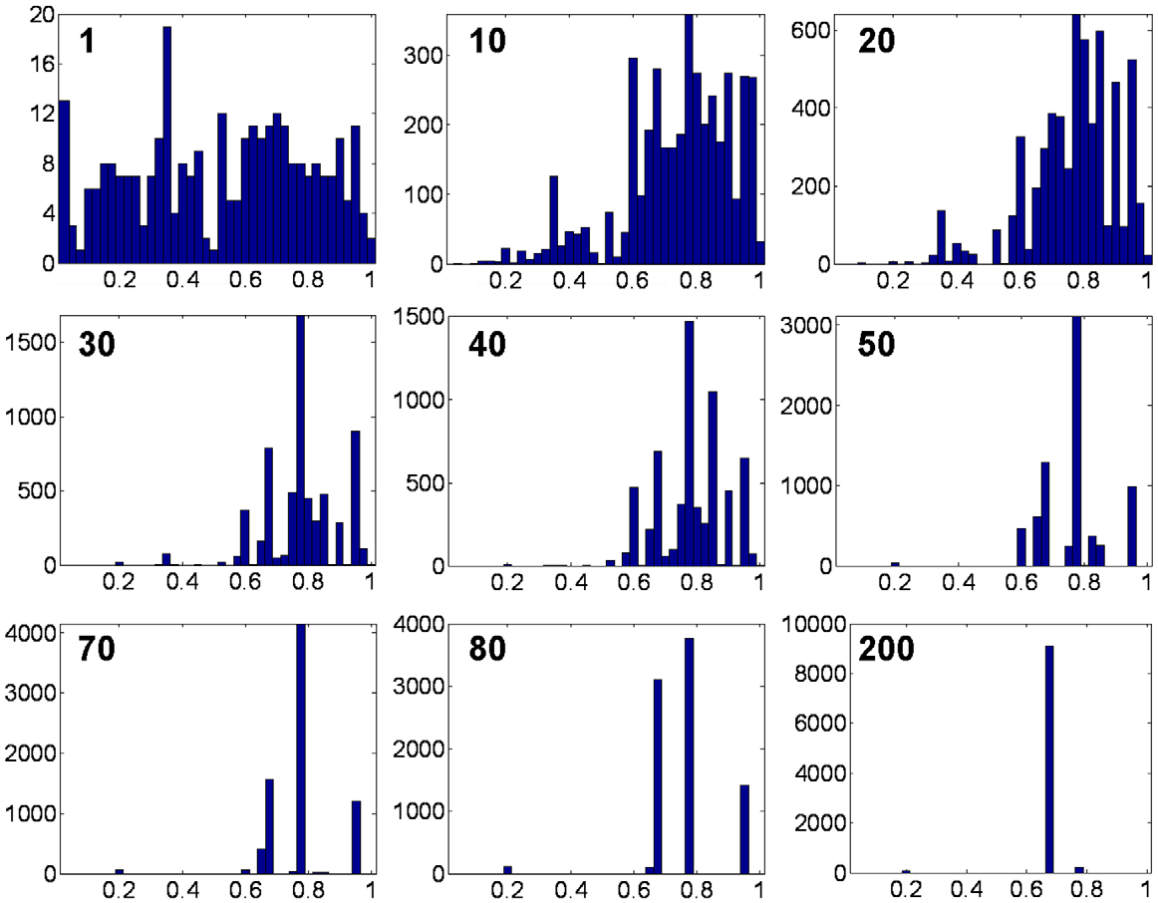
Distribution of values of μ in a competition experiment. The generation number is shown in bold face at the top of each histogram. The competition experiment shown here corresponds to experiment 8 in Figure 9 below.

**Figure 9:**
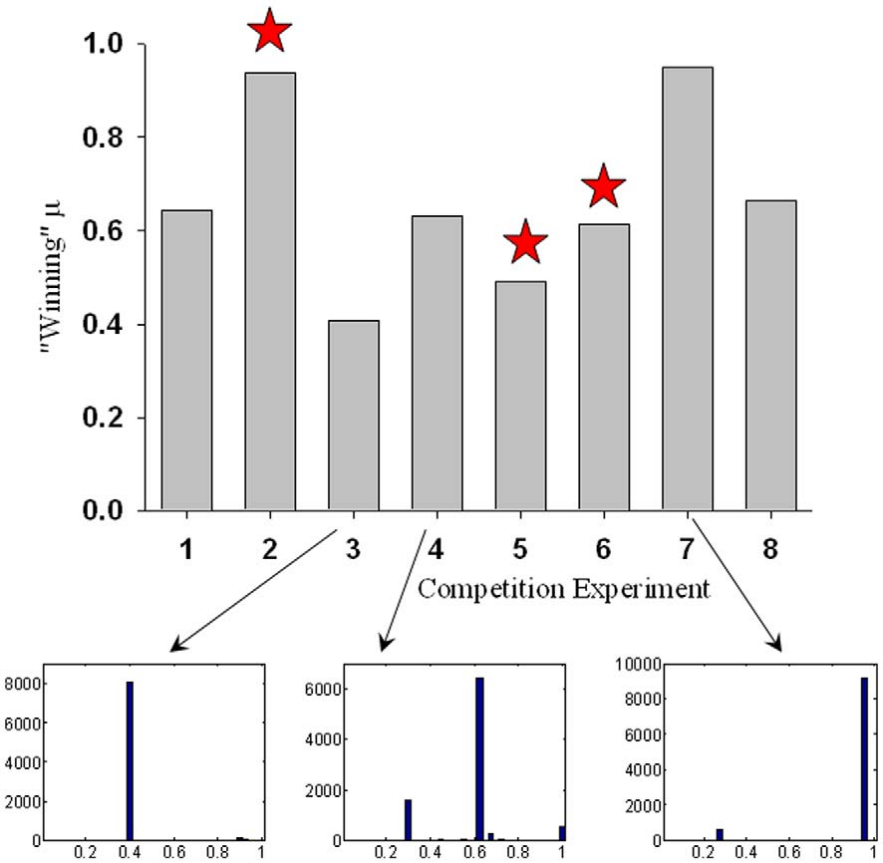
Distribution of values of surviving μ values after 500 generations for eight competition experiments. Stars indicate simulation runs (2, 5 and 6) where only a single value of μ survived. In other runs, most of the organisms at generation 500 exhibited a single value of μ, with smaller sub-populations exhibiting other μ values. Histograms of μ values for simulations 3, 4 and 7 are shown at the bottom of the figure as examples.

In Figure 8, we show a typical result of competition among organisms with different values of μ. The panels show distributions of μ throughout the population at various generations within a representative simulation. Initially, the distribution is uniform. (Note that since there are only 300 initial organisms, the uniform distribution is poorly sampled.) In the initial generations, most of the organisms with smaller values of μ become extinct. By generation 70, in this example, only a few values of μ remain represented in the population. These populations grow and shrink in size, jockeying for position in the fitness landscape, until, by generation 200, one value of μ dominates the population. In later generations (not shown), the other values of μ disappear entirely. The simulation illustrated in Figure 8 is typical; a single value of μ was always found to dominate the population after a number of generations. However, *different values of μ dominate in different runs of the simulation*, and the surviving μ (∼0.68 in the example shown in Figure 8) does *not* coincide with the value of μ (∼0.35) that gives a maximal number of species for the shifting landscape model with uniformly distributed mutations (Figure 3b) from which the competition simulation was derived. The variety of surviving μ values over various competition experiments is illustrated in Figure 9.

## Discussion

The model presented here illustrates that mutation size can affect the formation of clusters of organisms in a continuous morphospace. An intermediate maximum mutation size μ leads to a maximal number of clusters. It should be emphasized that the organisms in the model exist in a morphospace rather than a physical space, and thus the process of speciation modeled here is sympatric, rather than allopatric or parapatric.

In all implementations of the model, a high level of historical contingency was observed for intermediate values of the mutation parameter μ. This is illustrated in Figure 2, where, for identical conditions, two radically different outcomes are observed. The existence of contingent evolutionary outcomes has been discussed extensively by Gould [33], among others, and has recently been experimentally demonstrated by Lenski's group in their long term evolution experiment (LTEE) with populations of E. coli [34]. In the model presented here, such contingency is not observed for low values of μ, where populations exclusively tend towards extinction, or for high values of μ, where the mean population size has reached a plateau. Rather, contingent behavior occurs for the intermediate values of μ over which sharp changes in the system's behavior are observed: a sharp rise in population size and number of clusters, and a sharp drop in population-cluster correlation. The contingent behavior illustrated in Figure 2 is observed in all four versions of the model studied (data not shown), and always within a critical window of μ values.

As illustrated in Figures 3a, 4a, 5a and 6a, we observe a low mean population size for low values of μ, a sharp rise in population size for intermediate values of μ, and a plateau in the population size for high values of μ. This can be interpreted based on the overpopulation limit imposed in the model. For low μ, new organisms remain in tight clusters, unable to explore the landscape far beyond the locations of their parents. This results in overcrowding, followed by a decrease of the population and eventual extinction. For high μ, the population cannot grow indefinitely, constrained by both the overpopulation limit and the finite landscape size, which combine to play a role analogous to the carrying capacity in logistic population models.

The number of clusters (Figures 3b, 4b, 5b and 6b) undergoes a sharp rise for intermediate values of μ, but then reaches a maximum, rather than a plateau. This maximum can be qualitatively explained as follows. For the highest values of μ, organisms can experience very large mutational jumps away from the locations of their parents. Since other organisms, and thus other clusters, may exist nearby, these far-jumping organisms may venture so far from their parents' locations that they fall into the purview of other clusters, rather than either enlarging the cluster in which they originated, or nucleating a new cluster. This drives the total number of clusters lower as μ increases.

A measure of diversity within clusters shows a minimum for an intermediate value of μ (Figures 3c, 4c, 5c and 6c). This result might seem counterintuitive, since for low values of μ, the organisms cannot get very far from the locations of their parents, and thus one would expect the diversity to be lowest for smallest μ. However, note that for low values of μ the populations quickly become extinct, so the diversity values are averaged over a small number of generations. This means that the initial seeding of the landscape (at random locations drawn from a uniform distribution) contributes heavily to the diversity calculation. During early generations, organisms are forced to mate with partners which may be a considerable distance away, and therefore clusters will be less dense (more diverse). The diversity will reach a minimum for μ large enough that the population survives for sufficient generations to render the early “wide” clusters negligible in the diversity calculation. For values of μ beyond this point, diversity increases with μ, reaching a plateau as the mean population size approaches its asymptotic limit.

The population size and the number of clusters within each simulation correlate closely for small μ, as shown in Figures 3d, 4d, 5d and 6d. For values of μ near the critical transition to higher population size and maximum number of clusters, this correlation very suddenly becomes unpredictable. In some runs, a high correlation is observed; in others, there is essentially no correlation, and in still others, an anticorrelation. This can be interpreted in light of the different scenarios shown in Figure 2. For μ within the critical range (see, for example, μ = 0.9 in Figure 6, corresponding to the examples illustrated in Figure 2), the population can sometimes become extinct (Figure 2b). In this case, both population size and number of clusters tend sharply – and simultaneously – toward zero, and hence are strongly correlated. When the population does *not* become extinct, however, as in Figure 2c, population and clusters are not necessarily correlated, and indeed may be anticorrelated. This range of possible outcomes is the source of the large error bars around μ = 0.9 in Figure 6d.

For values of μ beyond this critical range, the population size and the number of clusters are strongly anticorrelated, which can be interpreted as follows. As the population increases for larger values of μ and the landscape becomes filled, new organisms will not be able to seed new clusters due to the finite size of the landscape: they will have nowhere to go except into some other cluster, and there will be no new possible niches they can colonize. This will effectively lead to the merging of clusters, and a decrease in the number of clusters.

The model studied here is remarkably robust across various modifications, including changes in how the landscape varies (gradual shift vs. feedback) and in the distribution of mutation sizes (uniform vs. normal). However, some differences do occur between the various implementations of the model. For example, the cases where mutation sizes are normally distributed (Figures 5 and 6) exhibit transitions in the parameters of interest for significantly higher values of μ than the cases where mutation sizes are uniformly distributed (Figures 3 and 4). This can be interpreted as follows. Mutations selected from a zero-mean normal distribution will tend to be small, with the majority being around size zero. Therefore, new organisms will not vary far from the locations of their parents for small values of μ, and, due to the overpopulation limit, will tend toward extinction (see, for example, Figures 5a and 6a for μ<0.65). For a uniform distribution of mutation values, however, the mutation values will be as likely to land the new organism near the limiting value of μ as they will be to leave them close to a point intermediate between their parents (mutation size = 0). Thus, a significant proportion of the organisms will be able to venture farther into the morphospace for smaller values of μ, as seen in Figures 3 and 4. Note that in the cases shown in Figures 5 and 6, an increase of the parameter *b* in Eq. (2) will increase the width of the normal distribution of mutation sizes, shifting the plots to the left.

A second difference among the different versions of the model concerns the sharpness in the drop of the correlation between population size and number of clusters. For the models without feedback (Figures 3d and 5d) the correlation decreases gradually. For the models including feedback, however (Figures 4d and 6d), the correlation drops far more sharply. This difference between the models can be explained as follows. Consider the anticorrelation between the population and the number of clusters as μ increases, discussed above. The feedback model strongly exacerbates this anticorrelation. For large values of μ, the organisms expand to fill the most advantageous regions of the morphospace (i.e., those where they will produce the most offspring). Increased μ allows them to take more advantage of these regions, since they can more easily spread through these areas. However, once they begin to flourish at a particular location, the feedback aspect of the model will render the regions less advantageous, leading to an ultimate decrease in population. Some of this population drop will lead to the splitting of formerly large clusters. Thus, as population decreases, the number of clusters will increase. Likewise, depopulated regions will increase in fitness, leading to an increase in population, and coalescence of smaller clusters; this again leads to an exaggerated anticorrelation between the two quantities. This effect of feedback, leading to negatively correlated fluctuations in both population and number of clusters over time, may also explain the greater variability in all quantities for the feedback models, visible in the much larger standard deviations in Figures 4 and 6 than in Figures 3 and 5.

For all versions of the model, sharp changes are observed in various quantities characterizing the system as μ is varied. These sharp changes are strongly reminiscent of phase transitions, with population size, number of clusters and population-cluster correlation serving as *order parameters* characterizing the system. Not only do these parameters exhibit sharp changes in their mean values as a function of μ, but they also exhibit large fluctuations (i.e., large standard deviations) during the transition, another characteristic of phase transitions. It is possible that further investigation of this and related models from such a statistical physics perspective may be of critical importance in understanding the role of mutation rate and mutation size in modulating speciation. Moreover, such models may eventually begin to touch upon a fundamental problem of evolutionary biology: the bridge between micro (individual) and macro (demes, species, genera) levels. The phase transition behavior observed here is particularly tantalizing in this regard, since a key characteristic of phase transitions is the *co-existence of multiple scales* of behavior. Further, in the model presented here, properties at the individual level (such as the parameter μ) affect the global dynamics of the entire population, both by modulating the formation of clusters, and modulating the average properties of these clusters. It should be emphasized that this relation *between scales* is fundamentally in the spirit of statistical physics, where microscopic dynamics determine global, macroscopic behavior.

If an intermediate mutation parameter maximizes the number of clusters, does this mean that this value of μ is optimal? The competition experiment illustrated in Figure 8 shows that a single value of μ tends to dominate the population in the limit of many generations. However, we find that the surviving value of μ varies from one run to the next, as illustrated in Figure 9. Given the irregularity and variability of the landscape, and seen in the light of the results of Earl and Deem [16] and Clune et al. [20], this is perhaps not surprising. The strong role of contingency, as well as the variability of the landscape and the fact that the system is not driven only to maximize the number of clusters, but is also subject to other pressures “from below”, such as a basic increase in population, all tend toward different values of μ surviving in different runs of the model.

It is striking that multiple stable populations with different values of μ have not been found to coexist in this model. However, the simulations did not allow for the reemergence of extinct mutation values, and thus perhaps the eventual dominance of a single value of μ may be inevitable, especially given the system's tendency in the limit of a large number of generations to exhibit a fully mixed population where all organisms share at least one common ancestor (as shown in Figure 7). Nonetheless, the fact that different values of μ survive in different realizations of this experiment strongly emphasizes not only the high degree of contingency in such systems, but also the delicate balance of various competing optimalities as a population struggles to expand into every available crevice of its morphospace.

## References

[1] Sturtevant AH (1937). Essays on Evolution I. On the effects of selection on mutation rate.. Q Rev Biol.

[2] Sniegowski PD, Gerrish PJ, Johnson T, Shaver A (2000). The evolution of mutation rates: separating causes from consequences.. BioEssays.

[3] Kimura M (1967). On the evolutionary adjustment of spontaneous mutation rates.. Genet Res.

[4] Fisher RA (1930). The Genetical Theory of Natural Selection.

[5] Crow JF (1997). The high spontaneous mutation rate: is it a health risk?. Proc Natl Acad Sci USA.

[6] Giraud A, Matic I, Tenaillon O, Clara A, Radman M, Fons M, Taddei F (2001). Costs and benefits of high mutation rates: adaptive evolution of bacteria in the mouse gut.. Science.

[7] Drake JW (1991). A constant rate of spontaneous mutation in DNA-based microbes.. Proc Natl Acad Sci USA.

[8] Drake JW, Charlesworth B, Charlesworth D, Crow JF (1998). Rates of spontaneous mutation.. Genetics.

[9] Drake JW, Allen EF, Forsberg SA, Preparata R-M, Greening EO (1969). Genetic control of mutation rates in bacteriophage T4.. Nature.

[10] Schapper RM (1998). Antimutator mutants in bacteriophage T4 and Escherichia coli.. Genetics.

[11] Nöthel H (1987). Adaptation of *Drosophila melanogaster* populations to high mutation pressure: Evolutionary adjustment of mutation rates.. Proc Natl Acad Sci USA.

[12] Sniegowski PD, Gerrish PJ, Lenski RE (1997). Evolution of high mutation rates in experimental populations of *E. coli*.. Nature.

[13] Denamur E, Matic I (2006). Evolution of mutation rates in bacteria.. Molec Microbiology.

[14] Pigliucci M (2007). Do we need an extended evolutionary synthesis?. Evolution.

[15] Pennisi E (2008). Modernizing the modern synthesis.. Science.

[16] Earl DJ, Deem MW (2004). Evolvability is a selectable trait.. Proc Natl Acad Sci USA.

[17] Bell G (2005). The evolution of evolution.. Heredity.

[18] Pigliucci M (2008). Is evolvability evolvable?. Nature Reviews Genetics.

[19] Bedau MA, Packard NH (2003). Evolution of evolvability via adaptation of mutation rates.. Biosystems.

[20] Clune J, Misevic D, Ofria C, Lenski RE, Elena SF, Sanjuán R (2008). Natural selection fails to optimize mutation rates for long term adaptation on rugged fitness landscapes.. PLoS Comput Biol.

[21] Kauffman SA (1993). The Origins of Order: Self-Organization and Selection in Evolution.

[22] Bak P (1996). How Nature Works: the Science of Self-Organised Criticality.

[23] Gould SJ (2002). The Structure of Evolutionary Theory.

[24] Kondrashov AS, Shpak M (1998). On the origin of species by means of assortative mating.. Proc Biol Sci.

[25] Pennings PS, Kopp M, Meszéna G, Dieckmann U, Hermisson J (2008). An analytically tractable model for competitive speciation.. Am Nat.

[26] Bürger R, Schneider KA, Willensdorfer M (2006). The conditions for speciation through intraspecific competition.. Evolution.

[27] de Cara MA, Barton NH, Kirkpatrick M (2008). A model for the evolution of assortative mating.. Am Nat.

[28] Otto SP, Servedio MR, Nuismer SL (2008). Frequency-dependent selection and the evolution of assortative mating.. Genetics.

[29] Kopp M, Hermisson J (2008). Competitive speciation and costs of choosiness.. J Evol Biol.

[30] Maynard Smith J, Szathmáry E (1995). The Major Transitions in Evolution.

[31] Mallet J (1995). A species definition for the modern synthesis.. Trends in Ecol Evol.

[32] de Aguiar MA, Baranger M, Baptestini EM, Kaufman L, Bar-Yam Y (2009). Global patterns of speciation and diversity.. Nature.

[33] Gould SJ (1989). Wonderful Life: the Burgess Shale and the Nature of History.

[34] Blount ZD, Borland CZ, Lenski RE (2008). Historical contingency and the evolution of a key innovation in an experimental population of *Escherichia coli*.. Proc Natl Acad Sci USA.

